# Socioeconomic inequality in health-related quality of life among Korean adults with chronic disease: an analysis of the Korean Community Health Survey

**DOI:** 10.4178/epih.e2024018

**Published:** 2024-01-08

**Authors:** Thi Huyen Trang Nguyen, Thi Tra Bui, Jinhee Lee, Kui Son Choi, Hyunsoon Cho, Jin-Kyoung Oh

**Affiliations:** 1 Department of Cancer Control and Population Health, Graduate School of Cancer Science and Policy, National Cancer Center, Goyang, Korea; 2 Department of Cancer AI & Digital Health, Graduate School of Cancer Science and Policy, National Cancer Center, Goyang, Korea

**Keywords:** Quality of life, Chronic disease, Inequality, Socioeconomic, Health inequalities

## Abstract

**OBJECTIVES:**

Health-related quality of life is crucial for people dealing with chronic illness. This study investigated the quality of life in individuals with 5 common chronic conditions in Korea. We also analyzed socioeconomic factors such as education, income, occupation, and urbanization to identify determinants of inequality.

**METHODS:**

Using 2016 Korea Community Health Survey data, we examined individuals aged 30 or older with chronic diseases (diabetes, hypertension, cardiovascular disease, hyperlipidemia, arthritis) using the EuroQol 5-Dimension 3 Level tool. We analyzed the associations between socioeconomic factors (education, income, occupation, urbanization) and quality of life using descriptive statistics and regression analysis. Inequality indices (relative inequality index, absolute inequality index) were used to measure inequality in quality of life.

**RESULTS:**

Individuals with higher income levels showed a 1.95-fold higher likelihood of a better quality of life than those with the lowest income. The lowest income group had higher odds of mobility (adjusted odds ratio [aOR], 2.2), self-care (aOR, 2.1), activity limitations (aOR, 2.4), pain/discomfort (aOR, 1.8), and anxiety/depression (aOR, 2.3). Educational disparities included a 3-fold increase in mobility and daily activity problems for those with elementary or lower education. Well-educated participants had a 1.94 times higher quality of life, with smaller differences in anxiety/depression and self-management. The income gap accounted for 14.1% of variance in quality-of-life disparities.

**CONCLUSIONS:**

Addressing socioeconomic disparities in the quality of life for individuals with chronic diseases necessitates tailored interventions and targeted health policies. This research informs policymakers in developing focused initiatives to alleviate health inequities. It emphasizes the importance of mental health support and ensuring affordable, accessible healthcare services.

## GRAPHICAL ABSTRACT

[Fig f3-epih-46-e2024018]

## Key Message

• Quality of life score (EQ-5D) in patients with chronic diseases (i.e., diabetes, hypertension, cardiovascular disease, hyperlipidemia, and arthritis) that are prevalent in Koreans identified through data from community health surveys averaged 0.7, lower in women than in men, and decreased by age.

• Low-income or low-educated patients have relatively low quality of life, and they have more than twice as much problem in mobility, self-care, pain/discomfort, and anxiety/depression.

• Disparities in quality of life in patients with chronic diseases according to socioeconomic conditions have been found, and support for the vulnerable is needed.

## INTRODUCTION

Health-related quality of life (HRQoL) is an essential indicator of overall health and well-being because it relates to an individual’s subjective physical and mental health. The unequal distribution of HRQoL among populations, particularly among people with chronic conditions, has become an increasing global concern, especially as the prevalence of chronic diseases such as diabetes, hypertension, cardiovascular diseases, and cancer has been rapidly increasing [1-3]. The unequal distribution of HRQoL among patients with chronic illnesses is a developing problem that is significantly affected by socioeconomic variables. Individuals with chronic diseases have lower HRQoL than healthy individuals; this inequality is often compounded by relevant socioeconomic factors, including income, education, and employment status [4,5].

Previous studies have investigated the unequal distribution of HRQoL among patients with chronic diseases. For example, a study conducted in Germany reported that among individuals with chronic diseases, those with lower education had lower HRQoL scores than those with higher education [6]. Similarly, a study conducted in the United States found that people with diabetes, heart disease, or stroke had lower HRQoL scores than those without these conditions; additionally, these scores were lower among individuals with lower income and education [7]. Another Australian study reported that people with chronic diseases such as arthritis, diabetes, and heart disease had lower HRQoL scores than those without these conditions; additionally, these scores were considerably lower in those with reduced income and education levels [8].

Patients with diabetes in rural areas were found to have lower HRQoL scores in environmental categories than those in urban areas [9]. Moreover, income and education levels were significantly associated with HRQoL among individuals with chronic diseases, and lower income and education levels were associated with poorer HRQoL scores [10].

In Korea, several studies have investigated the unequal distribution of HRQoL among patients with chronic diseases [11-14]. A study conducted among older adults with chronic diseases found that women or those with lower socioeconomic status (SES) had lower HRQoL scores than men or those with higher SES [11]. Additionally, a study investigating HRQoL among Korean adults showed that better HRQoL scores were more prevalent among young adults with higher income and education [12]. Similarly, unemployed patients with chronic kidney disease and lower SES reported worse health conditions and lower HRQoL than those with higher SES or employed patients [13]. Moreover, among cancer survivors in Korea, unemployment status or lower education was associated with lower HRQoL [15,16].

Earlier investigations predominantly centered on older individuals, concentrating either on 1 or 2 chronic diseases or employing small sample sizes. Furthermore, the prevailing trend in classifying SES primarily relied on educational attainment and individual income. Hence, there is a necessity for broader coverage within the population and the incorporation of diverse socioeconomic indicators. Additionally, while disparities in HRQoL indices have been documented, the understanding of the impact of each dimension on HRQoL remains limited. This research gap underscores the urgency of undertaking an in-depth study of this issue, especially given its potential to inform healthcare policy and possible healthcare services, including improvement in medication compliance and lifestyle habits to reduce health disparities and enhance HRQoL among people with chronic diseases. The primary objective of this study was to investigate the socioeconomic disparities in HRQoL among individuals dealing with chronic diseases. In addition to this overarching goal, the study aimed to identify the determinants that contribute to these socioeconomic discrepancies in HRQoL. Notably, the research also sought to discern the impact of such inequality on both the overall quality of life (QoL) and each dimension of HRQoL among patients with chronic diseases. The anticipated outcomes of this investigation are expected to provide valuable insights that can inform strategies aimed at mitigating disparities in HRQoL among people affected by chronic diseases.

## MATERIALS AND METHODS

### Data source and study subjects

We used data from the 2016 Korea Community Health Survey (CHS). This is a national cross-sectional survey conducted annually at 253 community health centers in Korea, with an average sample size of 900 people per district each year [16]. This survey was conducted to standardize community surveys for developing local district-level health initiatives, and contained 3 components: a household survey, an individual survey, and a common survey in administrative areas, which provided data on socioeconomic characteristics, health practices and behaviors, and common health issues [17]. This study specifically focused on the economically active population aged 30 years and older who had chronic diseases. The utilization of the CHS holds a distinct advantage due to its status as a nationally representative survey, contributing to the statistical stability of our findings.

### Study participants

The selection of households was the initial step. The *tong/ban/ri* (urban village, hamlet, and rural village, respectively) was chosen for various types of residences through probability sampling, considering its weight design. Household surveys were systematically chosen after counting households, based on the number of adults in the area [17]. On average, 5 households were sampled at each sampling point, and all household members aged ≥ 19 were interviewed. If there were no residents over 19 years old, the type of residence was not a household, or household members declined to participate or were absent, replacement households were selected. The analysis included participants > 30 years of age who completed the survey and reported any of the following 5 chronic diseases: hypertension, diabetes, stroke, angina pectoris/myocardial infarction, and hyperlipidemia or dyslipidemia.

### Health-related quality of life

The EuroQol 5-Dimension 3 Level (EQ-5D-3L) is a self-report survey measuring life quality across 5 domains: mobility, self-care, usual activities, pain/discomfort, and anxiety/depression [18]. Each dimension is scored on a 3-level severity ranking ranging from “no problems” to “extreme problems.” The EuroQol 5-Dimension (EQ-5D) utility was calculated based on the EQ-5D-3L value set of the Korean population [19,20].

### Socioeconomic status variables

We used the classification of the administrative districts provided by the 2016 CHS manual, which included 17 metropolitan cities and 258 districts. Based on administrative districts, urbanity was classified into 3 categories: rural areas (*gun* [county] local districts), metropolitan areas (*gu* [district] local districts), and urban areas (*si* [city] local districts). Cheongju-si, Cheonan-si, Jeonju-si, Changwon-si, Masan-si, and Jinhae-si were classified as metropolitan areas [21].

Educational attainment was classified into 3 categories: elementary or lower, middle, and high school, and university or higher. Using the Korean employment classification of occupations and the International Standard Classification of Occupations, which is based on the task/duties and skill level and skill specialization, we defined occupations into 5 groups, including professional managers/office workers; technical labor; sales and service/agriculture and fishery; housewives; and unemployed [22,23].

### Income quintile

The individual monthly income was calculated by dividing the total household income by the number of family members aged > 19 years. Income quintiles divide the population into 5 income divisions: the first quintile (bottom quintile): 0-20% of income; second quintile: 20-40% of income; third quintile: 40-60% of income; fourth quintile: 60-80% of income; and fifth quintile (top quintile): 80-100% of income. The income quintiles were analyzed by comparing the total income of the top 20% of the population (top quintile) to that of the bottom 20% (bottom quintile).

### Statistical analysis

We analyzed the distribution of chronic diseases and 5 dimensions of QoL by SES using descriptive statistics.

The individual weighting considered the personal response rate and household weighting. Correction weighting was utilized for estimation by adjusting individual weighting based on gender and the registered population’s age structure in each survey area [24].

We used the chi-square test to evaluate differences in the distribution of chronic diseases by urbanity, gender, and age group. The weighted mean and 95% confidence interval (CI) of EQ-5D utilities were provided for participants with chronic diseases. The significance of differences in weighted mean EQ-5D index score by socioeconomic characteristics was determined by the t-test and one-way analysis of variance. To determine the association between participants’ QoL and the number of chronic diseases, we conducted multivariate logistic regression analysis between the 5 dimensions of HRQoL and SES factors (educational attainment, occupation, and income) adjusted by age and gender. Additionally, multivariate linear regression models adjusted for age and gender were used to investigate the correlations between the EQ-5D utility score and chronic diseases.

### Inequality indices

Regression-based inequality and relative inequality index (RII) measures were calculated to investigate potential inequalities [25]. We applied Poisson regression analysis to obtain the RII by treating the EQ-5D index and 5 dimensions of HRQoL as indicators of the cumulative relative position of each group concerning education and income levels. The RII can be interpreted as the risk ratio. Therefore, the results can reflect relative disparities. An RII value of 1 shows that there is no inequality in the prevalence of low QoL, whereas a RII value larger than 1 implies a higher chance of having low QoL. The absolute inequality index (SII) was also calculated. An SII value > 1 indicates a difference in low QoL prevalence between the highest and lowest socioeconomic groups, which is presented as a percentage.

All analyses were applied to the survey weights adjusted for study design with consideration of strata. A p-value < 0.05 was considered to indicate statistical significance.

### Ethics statement

The CHS surveys were approved by the Research Ethics Review Committee of the Korea Centers for Disease Control and Prevention (2010-02CON-22-P; 2012-07CON-01-2C; 2014-08EXP-09-4C-A; 2016-10-01-P-A). This study was exempted from review by the Institutional Review Board of the National Cancer Center, Korea (NCC2023-0114).

## RESULTS

### Characteristics of the study population

We enrolled 84,581 participants with chronic diseases aged ≥ 30 years in the study. Table 1 shows the average score of EQ-5D utility. Overall, the mean score of the EQ-5D index was 0.7, and men participants reported higher utility scores than women (0.81 and 0.59, respectively). Younger individuals had better QoL (0.90 among those 30-55 years of age) than older individuals (0.75 among those 56-70 years of age; 0.37 among those > 70 years of age) in both genders. Men respondents with higher income (0.92 in Q5) reported higher utility scores of QoL than those in lower income groups (0.51 in Q1). Participants with lower education (0.42 in those with an elementary or lower education), unemployed individuals (0.48), and those living in rural areas (0.65) reported worse weighted mean EQ-5D scores than those with higher education (0.90 in those with a university or higher education), those who worked as professional managers/office workers (0.94), and those who lived in metropolitan areas (0.73).

**Figure 1.**
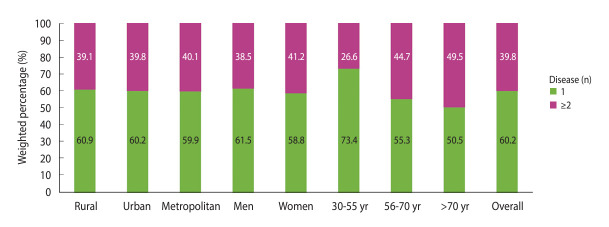
Community-level rates of unhealthy living, stratified by gender, age group, and chronic health conditions.

Of all respondents, 39.8% had at least 2 chronic diseases (Figure 1). The proportion of individuals having more than 1 chronic disease varied across age groups (chi-square p-value< 0.05) and was highest among the 56-70 and >70 age groups (44.7 and 49.5%, respectively).

Table 2 presents the proportion of individuals who reported problems with various EQ-5D dimensions, including mobility, self-care, activity, pain/discomfort, and anxiety/depression. The highest proportion of problems reported by participants was pain/discomfort, which accounted for 38.5% (95% CI, 38.0 to 38.9). Older respondents with lower income or education were more likely to report more problems in the 5 dimensions of HRQoL, while those who lived in metropolitan areas reported fewer problems than those who lived in rural areas. Among individuals with a history of stroke, 59.3% reported problems related to mobility, signifying challenges in their ability to move around (Table 2). Additionally, 36.8% faced self-care difficulties, indicating the lingering impact of stroke on their daily activities. For individuals with diabetes, 30.4% faced problems with mobility, suggesting potential issues in their physical functioning due to this chronic condition, and around 12.2% encountered difficulties in self-care. Regarding hyperlipidemia, 21.0% of respondents indicated problems with mobility. Among individuals with general cardiovascular conditions, 39.6% reported mobility-related problems, and 16.1% encountered self-care issues. Among individuals with more than 1 chronic disease, 31.9% experienced problems with mobility and 12.7% faced self-care challenges, indicating potential complexities in managing multiple health issues simultaneously. Of 84,581 respondents, 26,452 (30.5%) reported a negative utility score, and 40,442 (69.5%) reported a full EQ-5D index (data not shown). After adjusting for all variables, our results showed that older participants and those living in rural/urban areas were more likely to report problems in mobility, self-care, and activity (Table 3). Individuals aged > 70 were 3.2 times (95% CI, 2.9 to 3.5) more likely to report problems in mobility, 1.9 times (95% CI, 1.6 to 2.2) more likely to have self-care problems, and 2.2 times (95% CI, 2.0 to 2.4) more likely to report problems in usual activities (Table 3) than the youngest group (30-55 years). Similarly, residents living in rural and urban areas were also significantly less likely to report pain/discomfort and anxiety/depression than those living in metropolitan areas. The regression results showed that the mean EQ-5D index score was 0.15 lower among individuals with the lowest income compared to those with the highest income. Additionally, it was 0.24 lower among those with an elementary education compared to those with a university or higher education (Table 3).

### Absolute and relative inequality in quality of life among patients with chronic diseases

**Figure 2.**
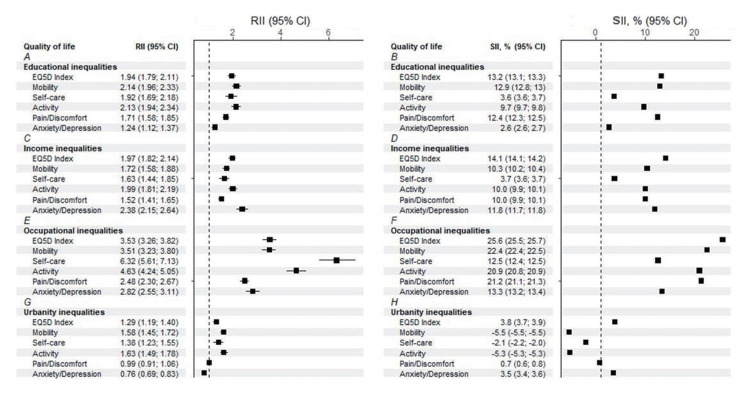
Absolute and relative inequality in the quality of life (EuroQol 5-Dimension [EQ-5D] index) and quality of life dimensions. A, B: Relative and absolute educational inequalities in quality of life. C, D: Relative and absolute income inequalities in quality of life. E, F: Relative and absolute occupational inequalities in quality of life. G, H: Relative and absolute urbanity inequalities in quality of life. RII, relative index of inequality; SII, absolute inequality index; CI, confidence interval.

Figure 2 shows the socioeconomic inequality in QoL. The difference in QoL between the highest and lowest education levels accounted for 13.2% of variance in educational attainment. Furthermore, people with the highest levels of education were 1.94 times more likely to have better QoL (RII, 1.94; 95% CI, 1.79 to 2.11; Figure 2A). We also found significant educational inequality in the 5 dimensions of QoL. Participants with higher education were more likely to report better QoL and more likely to experience better well-being. Even the smaller differences in anxiety/depression and self-care favored those with university or higher education (anxiety/depression: RII, 1.24; SII, 2.6%; self-care: RII, 1.92; SII, 3.6%; Figure 2A and B).

Regarding income inequality, statistically significant results were found in both SII and RII. The EQ-5D index exhibited an RII of 1.97 (95% CI, 1.82 to 2.14) and an SII of 14.1% (95% CI, 14.1 to 14.2; Figure 2C and D). Consistently, significant results were found in 4 dimensions of QoL, including mobility (RII, 1.72; SII, 10.3%), activity (RII, 1.99; SII, 10.0%), pain/discomfort (RII, 1.52; SII, 10.0%), and anxiety/depression (RII, 2.38; SII, 11.8%). The difference between the highest and lowest income in self-care was 3.66%.

Occupational inequality is described in Figure 2E and F. The difference in QoL was especially large between those who were managers and those who were unemployed. Regarding absolute inequality, the difference in QoL between the manager and unemployed groups reached 25.6% (Figure 2E), and managers were 3.53 times more likely to have better QoL.

Regarding urbanity, relative and absolute inequalities were significant in EQ-5D utility scores, mobility, self-care, and daily activity (Figure 2G and H). Individuals residing in rural regions had a higher risk of low QoL than those living in metropolitan areas, with an RII of 1.29 (95% CI, 1.19 to 1.40) and an SII of 3.8% (95% CI, 3.7 to 3.9). Furthermore, we found that significant relative and absolute inequalities in mobility (RII, 1.58; SII, -5.5%), self-care (RII, 1.38; SII, -2.1%), activity (RII, 1.63; SII, -5.3%) favored people living in metropolitan areas. However, people living in rural areas were 0.76 times less likely to have problems with anxiety/depression, and the prevalence of anxiety/depression among metropolitan residents was 3.5% higher than among rural residents (Figure 2H).

## DISCUSSION

Our findings reveal that individuals with chronic diseases who had higher SES experienced higher utility scores of QoL and fewer problems in the 5 dimensions of QoL. However, the overall socioeconomic discrepancy in HRQoL may differ from the inequality in each individual QoL dimension. Participants with a low income had lower HRQoL than those with a high income. Additionally, people with a lower educational level had lower HRQoL than those with higher educational levels, even after adjusting for covariates. Our findings suggested that age, income, and education interact with chronic illness and HRQoL.

The study’s results are consistent with prior research reporting that socioeconomically disadvantaged groups had a higher frequency of chronic illness and low QoL. Chronic diseases are associated with unhealthy lifestyles [26], unsafe working environments, unhealthy living circumstances, difficulty accessing health care [27], late diagnoses, ineffective preventative medicine, and a higher incidence of comorbidities [28]. Among the Chinese healthy population, individuals in higher SES groups experienced higher utility scores of QoL than those in lower SES groups; in other words, low income and education levels were associated with poorer HRQoL [29]. The disparity in healthy lifestyle rates between urban/metropolitan cities and rural areas is a well-known issue in Korea. Compared with residents in rural areas, those who live in urban areas were found to engage more frequently in physical activity and were more likely to have a healthier diet [30]. Furthermore, healthcare costs related to disease management increase psychological distress, which is consistent with our results of lower scores on the psychological dimension of QoL among poorer individuals with chronic illnesses (i.e., those who are economically disadvantaged) [31]. Even healthcare services are highly accessible in Korea because of universal health coverage.

Our finding indicated that women had lower HRQoL than men in the social and environmental domains. A potential reason for this is that women are more likely to experience discrimination and gender-based violence, leading to poorer mental health and lower QoL [32-34]. Moreover, women are also more likely to be in lower-paid jobs, and have less job security and less access to economic resources, which can negatively impact their QoL [35]. Other factors impacting women’s QoL include lower levels of education and higher levels of caregiving responsibilities, which can cause stress and limit social participation [33].

Regarding the educational inequalities in HRQoL in our sample, we found that individuals with lower income and education had lower HRQoL than those in higher socioeconomic groups. This might be due to people with low SES having less access to healthcare, less healthy lifestyle behaviors, and higher stress levels, all of which can contribute to lower HRQoL [31,36]. Moreover, the low QoL of individuals with low income and education may be influenced by psychosocial factors such as stress, anxiety, and depression. Research has shown that people with lower SES often face higher levels of chronic stress due to financial insecurity, job insecurity, and social isolation [37]. This chronic stress can negatively impact physical and mental health, reducing QoL.

In addition to educational inequalities, we also investigated the income and occupational inequalities on HRQoL. Previous studies have reported an association between income levels and HRQoL, indicating a lower QoL among deprived groups [38]. Our results are consistent with those of previous studies, which suggest that low-income individuals have lower QoL and are more likely to have health problems. In Korea, even though National Health Insurance is mandatory for the entire population, we observed persistent income inequalities, which suggests that differences in economics should be addressed in health policies developed for low-income people to achieve better healthcare.

In line with previous research, our study identified a substantial association between residency (specifically categorizing individuals as residing in rural or metropolitan areas) and EQ-5D utility scores, particularly within the physical domain, encompassing aspects such as mobility, daily activities, and self-care. A significantly lower level of HRQoL was reported in the physical domain among residents in rural areas [35]. Notably, our results showed a higher risk of anxiety/depression among metropolitan residents than among those in rural areas. A previous study in China explained that individuals living in large cities may face multiple health risks, such as air pollution [39], noise, or overcrowded space [40]. Our study results provided more insight into the Korean context regarding the inequality between rural and metropolitan areas in terms of QoL utility scores and specific dimensions. However, individuals living in large cities were proven to have better healthcare access. Moreover, these individuals were more likely to engage in physical activity, including walking or cycling, which might improve their mental health [41,42].

The study findings showed that older participants reported lower QoL than younger participants, with a higher prevalence of problems in all 5 domains of HRQoL [36,43]. Herein, we included middle-aged and older participants who had difficulties meeting their healthcare needs [43]. The rate of unmet healthcare needs due to financial barriers among older adults aged > 65 was almost double that in the general population (2.6% among individuals aged above 65) [44]. However, after adjusting for covariate variables, our model showed a lower risk of anxiety/depression among older adults than among younger individuals. This can be explained by the fact that older people might accept the presence of their unhealthy conditions. Younger individuals tend to be part of working age groups; hence, their illness would have negative economic effects [45]. Chronic illnesses are associated with poorer employment engagement. Early retirement, working limits, and lower return to work influence SES and HRQoL [46].

This study possesses 2 notable strengths: a large sample size and the utilization of a validated measuring method for evaluating QoL. Unlike previous Korean studies that primarily focused on the overall utility scores of HRQoL, this study went a step further by delving deeper into each dimension of HRQoL. Therefore, it aimed to thoroughly understand how socioeconomic variables influence QoL overall and within each component. However, the limitations of this study should be acknowledged as well. First, while more recent data are available, the analysis was based on data from 2016. We chose these data because they cover a broader range of chronic conditions than previous survey years, boosting its comprehensiveness. Additionally, our study is limited by the exclusion of other chronic diseases that could potentially have a significant impact on HRQoL. Future research efforts should consider a more extensive range of chronic conditions to provide a more comprehensive evaluation. Third, the cross-sectional nature of the data precludes causal conclusions about the relationships between socioeconomic characteristics and QoL. Hence, longitudinal research is necessary to gain a more nuanced understanding of these connections. Finally, it is critical to note that the study was based on self-reported data, susceptible to response and social desirability biases.

In conclusion, our research demonstrates that socioeconomic factors, such as income and education, have a significant impact on various aspects of QoL, particularly in the physical, psychological, social, and environmental domains. Individuals with lower income and education levels experienced lower QoL, particularly in the physical, psychological, social, and environmental domains. These disparities may be exacerbated by limited healthcare access, unhealthy behaviors, and persistent stress. Therefore, our study underscores the importance of developing targeted healthcare interventions and policies aimed at addressing the specific needs of socioeconomically disadvantaged populations. Such measures are essential for mitigating QoL inequities, improving overall well-being, and reducing health disparities.

## Figures and Tables

**Figure f3-epih-46-e2024018:**
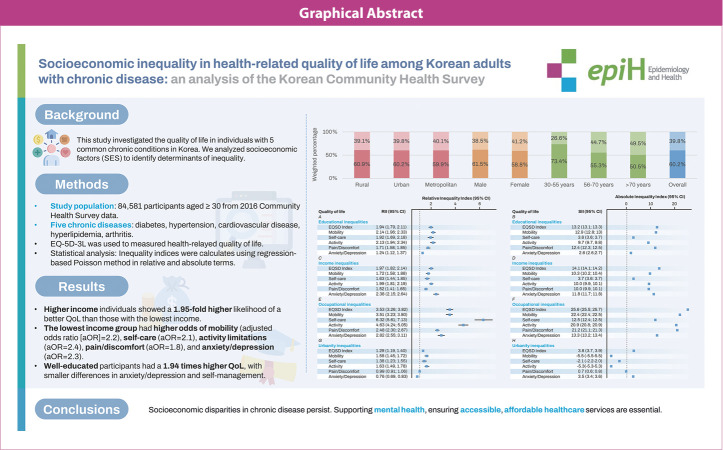


**Table 1.** Mean^1^ EuroQol 5-Dimension (EQ-5D) index scores by socio-demographic characteristics of the study sample

Characteristics	Total		Men		Women		p-value^2^
Overall	84,581 (100)	0.70 (0.70, 0.71)	46,755 (50.3)	0.81 (0.80, 0.81)	36,826 (48.9)	0.59 (0.59, 0.60)	<0.001
Age (yr)							
30-55	20,034 (33.9)	0.90 (0.89, 0.90)	11,438 (41.9)	0.92 (0.91, 0.92)	8,596 (25.7)	0.86 (0.85, 0.87)	<0.001
56-70	34,346 (40.1)	0.75 (0.75, 0.76)	15,426 (37.9)	0.83 (0.82, 0.84)	18,920 (42.3)	0.69 (0.68, 0.70)	<0.001
>70	30,201 (26.0)	0.37 (0.36, 0.37)	10,962 (20.2)	0.53 (0.52, 0.54)	19,239 (32.0)	0.26 (0.25, 0.27)	<0.001
Income quintiles							
Q1	28,678 (22.0)	0.40 (0.39, 0.41)	9,496 (15.7)	0.51 (0.49, 0.52)	19,182 (28.5)	0.34 (0.32, 0.35)	<0.001
Q2	16,985 (17.9)	0.66 (0.65, 0.67)	7,734 (16.5)	0.73 (0.72, 0.75)	9,251 (19.4)	0.59 (0.58, 0.60)	<0.001
Q3	13,076 (17.1)	0.78 (0.77, 0.79)	6,530 (18.0)	0.85 (0.84, 0.86)	6,546 (16.2)	0.70 (0.68, 0.71)	<0.001
Q4	15,646 (25.0)	0.83 (0.82, 0.84)	8,447 (28.6)	0.90 (0.89, 0.91)	7,199 (21.2)	0.74 (0.72, 0.75)	<0.001
Q5	9,453 (18.0)	0.87 (0.86, 0.87)	5,267 (21.2)	0.92 (0.91, 0.93)	4,186 (14.7)	0.79 (0.77, 0.80)	<0.001
Education							
Elementary or lower	38,137 (30.6)	0.42 (0.41, 0.43)	10,116 (16.3)	0.56 (0.55, 0.58)	28,021 (45.2)	0.37 (0.36, 0.37)	<0.001
Middle and high school	33,392 (45.1)	0.79 (0.78, 0.79)	18,324 (48.1)	0.81 (0.80, 0.82)	15,068 (41.9)	0.76 (0.75, 0.77)	<0.001
University or higher	12,926 (24.4)	0.90 (0.89, 0.91)	9,320 (35.5)	0.91 (0.91, 0.92)	3,606 (12.9)	0.87 (0.85, 0.88)	<0.001
Occupational							
Professional manager/Office worker	8,462 (16.6)	0.94 (0.93, 0.94)	6,216 (25.2)	0.94 (0.94, 0.95)	2,246 (7.7)	0.91 (0.90, 0.92)	<0.001
Technical labor	14,799 (20.5)	0.86 (0.85, 0.86)	9,424 (29.2)	0.90 (0.89, 0.90)	5,375 (11.9)	0.75 (0.74, 0.77)	<0.001
Sales and service/Agriculture and fishery	22,370 (17.3)	0.78 (0.78, 0.79)	10,278 (16.5)	0.84 (0.83, 0.85)	12,092 (18.2)	0.73 (0.72, 0,74)	<0.001
Housewife	20,521 (24.5)	0.55 (0.54, 0.56)	77 (0.2)	0.65 (0.52, 0.78)	22,444 (49.4)	0.55 (0.54, 0.56)	<0.001
Unemployed	18,367 (21.0)	0.48 (0.46, 0.49)	11,797 (29.0)	0.57 (0.56, 0.59)	6,570 (12.9)	0.25 (0.23, 0.27)	<0.001
Urbanity							
Rural	37,574 (20.3)	0.65 (0.65, 0.66)	15,913 (19.5)	0.78 (0.77, 0.79)	21,661 (21.0)	0.53 (0.52, 0.54)	<0.001
Urban	17,532 (18.6)	0.67 (0.66, 0.67)	7,957 (18.6)	0.78 (0.77, 0.79)	9,575 (18.7)	0.55 (0.53, 0.54)	<0.001
Metropolitan	29,475 (61.1)	0.73 (0.72, 0.73)	13,956 (61.9)	0.82 (0.81, 0.83)	15,519 (60.3)	0.63 (0.62, 0.64)	<0.001

Values are presented as number (%) or mean (95% confidence interval); All values were calculated by applying sampling weights assigned to individual participants in the survey.

1 Weighted mean, adjusted for survey design (strata, primary sampling unit) (95% confidence interval) of the individual EQ-5D utility score, stratified by gender.

2 The p-value was estimated for the mean difference between men and women, and a p-value <0.05 indicates statistical significance.

**Table 2.** Distribution of having problems on the EuroQol 5-Dimension (EQ-5D) scale by socio-demographic characteristics^1^

Variables^2^	Mobility	Self-care	Activity	Pain/Discomfort	Anxiety/Depression
Gender					
Men	15.4 (14.9, 15.8)	6.1 (5.8, 6.4)	12.2 (11.8, 12.6)	27.5 (26.9, 28.1)	12.5 (12.0, 12.9)
Women	32.2 (32.6, 33.7)^***^	12.0 (11.6, 12.3)^***^	25.6 (25.0, 26.1)^***^	49.8 (49.2, 50.4)^***^	23.1 (22.6, 23.6)^***^
Age (yr)					
30-55 (reference)	6.9 (6.5, 7.3)	2.3 (2.1, 2.6)	5.5 (5.1, 5.8)	24.0 (23.4, 24.7)	13.1 (12.6, 13.7)
56-70	19.5 (18.9, 20.0)^*^	5.8 (5.5, 6.1)^***^	14.4 (14.0, 14.9)^**^	36.1 (35.4, 36.7)^***^	16.5 (15.9, 17.0)^***^
>70	54.0 (53.3, 54.7)^***^	22.7 (22.1, 23.3)^***^	42.9 (42.2, 43.6)^***^	61.0 (60.3, 61.7)^***^	25.5 (24.9, 26.1)^***^
Income quintiles					
Q1 (reference)	50.3 (49.6, 51.1)	20.7 (20.1, 21.3)	41.5 (40.8, 42.2)	60.4 (59.7, 61.1)	29.8 (29.1, 30.5)
Q2	28.1 (27.3, 28.8)^***^	9.8 (9.3, 10.3)^***^	21.3 (20.6, 22.0)^***^	43.0 (42.2, 43.9)^***^	19.3 (18.7, 20.0)^***^
Q3	17.6 (16.9, 18.3)^***^	5.7 (5.3, 6.1)^***^	12.9 (12.3, 13.5)^***^	33.4 (32.5, 34.2)^***^	15.2 (14.5, 16.0)^***^
Q4	13.0 (12.4, 13.5)^***^	4.6 (4.3, 5.0)^***^	9.5 (9.0, 10.0)^***^	29.1 (28.3, 29.9)^***^	12.3 (11.7, 12.9)^***^
Q5	10.0 (9.4, 10.6)^***^	3.1 (2.8, 3.5)^***^	7.1 (6.6, 7.6)^***^	25.2 (24.2, 26.1)^***^	10.9 (10.2, 11.6)^***^
Education					
Elementary or lower (reference)	48.9 (48.2, 49.6)	19.2 (18.7, 19.7)	38.7 (38.0, 39.4)	59.7 (59.0, 60.3)	25.5 (24.9, 26.1)
Middle and high school	16.7 (16.2, 17.2)^***^	5.6 (5.3, 5.9)^***^	12.8 (12.3, 13.2)^***^	33.3 (32.6, 33.9)^***^	15.9 (15.4, 16.4)^***^
University or higher	7.0 (6.6, 7.5)^***^	2.5 (2.2, 2.8)^***^	5.0 (4.6, 5.4)^***^	21.6 (20.8, 22.4)^***^	11.3 (10.6, 11.9)^***^
Occupational					
Professional manager/Office worker (reference)	4.1 (3.7, 4.5)	0.9 (0.7, 1.1)	2.4 (2.0, 2.7)	18.2 (17.3, 19.0)	8.9 (8.2, 9.5)
Technical labor	10.8 (10.3, 11.4)^***^	2.2 (2.0, 2.5)	7.0 (6.5, 7.4)^***^	27.5 (26.7, 28.4)^***^	11.5 (10.9, 12.1)^***^
Sales and service/Agriculture and fishery	17.1 (16.6, 17.7)^***^	4.1 (3.8, 4.3)^***^	12.2 (11.7, 23.6)	33.6 (32.9, 34.4)^***^	13.9 (13.3, 14.6)
Housewife	37.2 (36.5, 38.0)^***^	13.0 (12.4, 13.3)^***^	29.0 (28.3, 29.7)^***^	53.3 (52.5, 54.1)^***^	24.7 (24.1, 25.4)^***^
Unemployed	43.7 (42.9, 44.6)^***^	21.7 (20.8, 22.3)^***^	37.0 (36.2, 37.9)^***^	52.1 (51.2, 53.0)^***^	25.6 (24.8, 26.4)^***^
Urbanity					
Rural (reference)	28.7 (28.0, 29.4)	11.0 (10.6, 11.5)	22.8 (22.2, 23.4)	40.3 (39.5, 41.1)	16.8 (16.1, 17.4)
Urban	27.8 (26.9, 28.6)^***^	10.2 (9.6, 10.7)^***^	22.1 (21.3, 22.8)^***^	39.1 (38.2, 40.0)	17.2 (16.5, 17.9)
Metropolitan	21.6 (21.0, 22.1)^***^	8.0. (7.7, 8.3)^***^	16.5 (16.0, 17.0)^***^	37.0 (37.0, 38.3)^***^	18.1 (17.6, 18.6)^***^
Chronic disease (reference=0)					
Stroke	59.3 (57.9, 60.5)^*^	36.8 (35.6, 38.0)^*^	52.1 (50.8, 53.4)^*^	12.9 (12.1, 13.8)^*^	5.3 (4.7, 5.9)^*^
Diabetes	30.4 (29.7, 31.1)^*^	12.2 (11.7, 12.7)^*^	24.2 (23.5, 24.8)^*^	5.9 (5.6, 6.3)^*^	1.9 (1.7, 2.2)
Hypertension	27.7 (27.2, 28.1)^*^	10.5 (10.2, 10.8)^*^	21.5 (21.1, 21.9)^*^	4.9 (4.8, 5.2)	1.6 (1.4, 1.7)
Hyperlipidemia	21.0 (20.5, 21.5)	6.9 (6.6, 7.2)	16.1 (15.6, 16.6)	3.9 (3.7, 4.2)^*^	1.6 (1.5, 1.8)^*^
Cardinal	39.6 (38.5, 40.6)^*^	16.1 (15.3, 16.9)^*^	32.6 (31.6, 33.7)^*^	8.6 (8.0, 9.2)^*^	3.2 (2.9, 3.6)^*^
Presence of multiple chronic diseases	31.9 (31.3, 32.6)^*^	12.7 (12.2, 13.1)^*^	25.3 (24.7, 25.9)^*^	6.2 (5.8, 6.5)^*^	2.2 (2.0, 2.4)^*^

Values are presented as % (95% confidence interval); All values were calculated by applying sampling weights assigned to individual participants in the survey.

1 Weighted prevalence, adjusted for survey design (strata, primary sampling unit) (95% confidence interval) of individuals who reported problems in EQ-5D dimensions.

2 The first category of each demographic variable was selected as a reference for the univariate logistic regression.

* p<0.05,

** p<0.01,

*** p<0.001.

**Table 3.** Association of socio-demographic factors with having problems with EuroQol 5-Dimension (EQ-5D) and utility scores

Variables	aOR (95% CI)^1^					β (95% CI)^2^
	Mobility	Self-care	Activity	Pain/Discomfort	Anxiety/Depression	EQ-5D Index
Gender						
Men	1.0 (reference)	1.0 (reference)	1.0 (reference)	1.0 (reference)	1.0 (reference)	1.00 (reference)
Women	2.0 (1.9, 2.1)	1.7 (1.5, 1.8)	1.7 (1.6, 1.8)	1.9 (1.8, 2.0)	1.9 (1.7, 2.0)	-0.09 (-0.10, -0.09)
Age (yr)						
30-55	1.0 (reference)	1.0 (reference)	1.0 (reference)	1.0 (reference)	1.0 (reference)	1.00 (reference)
56-70	1.3 (1.2, 1.4)	0.9 (0.8, 1.0)	1.1 (1.0, 1.2)	1.0 (0.9, 1.0)	0.7 (0.7, 0.8)	0.01 (0.00, 0.01)
>70	3.2 (2.9, 3.5)	1.9 (1.6, 2.2)	2.2 (2.0, 2.4)	1.7 (1.5, 1.8)	0.8 (0.7, 0.9)	-0.20 (-0.21, -0.19)
Income quintiles						
Q1	2.2 (2.0, 2.4)	2.1 (1.8, 2.4)	2.4 (2.1, 2.6)	1.8 (1.7, 1.9)	2.3 (2.1, 2.6)	-0.15 (-0.16, -0.14)
Q2	1.5 (1.4, 1.7)	1.6 (1.4, 1.9)	1.6 (1.4, 1.8)	1.3 (1.2, 1.4)	1.6 (1.5, 1.8)	-0.04 (-0.05, -0.03)
Q3	1.2 (1.1, 1.4)	1.3 (1.1, 1.5)	1.3 (1.1, 1.5)	1.1 (1.0, 1.2)	1.3 (1.2, 1.5)	-0.01 (-0.02, 0.00)
Q4	1.1 (1.0, 1.3)	1.3 (1.1, 1.5)	1.2 (1.0, 1.3)	1.1 (1.0, 1.2)	1.1 (1.0, 1.2)	-0.01 (-0.01, 0.00)
Q5	1.0 (reference)	1.0 (reference)	1.0 (reference)	1.0 (reference)	1.0 (reference)	1.00 (reference)
Education						
Elementary or lower	3.0 (2.7, 3.4)	2.4 (2.1, 2.8)	3.0 (2.7, 3.4)	2.1 (2.0, 2.3)	1.3 (1.2, 1.5)	-0.24 (-0.25, -0.23)
Middle and high school	1.5 (1.4, 1.7)	1.4 (1.2, 1.6)	1.6 (1.4, 1.8)	1.3 (1.2, 1.4)	1.1 (1.0, 1.2)	-0.05 (-0.06, -0.05)
University or higher	1.0 (reference)	1.0 (reference)	1.0 (reference)	1.0 (reference)	1.0 (reference)	1.00 (reference)
Occupational						
Professional manager/Office worker	1.0 (reference)	1.0 (reference)	1.0 (reference)	1.0 (reference)	1.0 (reference)	1.00 (reference)
Technical labor	1.1 (0.9, 1.3)	1.1 (0.8, 1.5)	1.2 (1.0, 1.5)	1.1 (1.0, 1.2)	1.1 (0.9, 1.2)	0.03 (0.03, 0.04)
Sales and service/Agriculture and fishery	1.4 (1.2, 1.6)	1.6 (1.2, 2.1)	1.7 (1.4, 2.1)	1.2 (1.1, 1.3)	1.2 (1.0, 1.3)	0.00 (-0.01, 0.00)
Housewife	2.1 (1.8, 2.4)	3.2 (2.4, 4.3)	2.9 (2.4, 3.5)	1.5 (1.3, 1.6)	1.5 (1.4, 1.7)	-0.13 (-0.14, -0.12)
Unemployed	3.2 (2.8, 3.7)	6.4 (4.8, 8.5)	4.8 (4.0, 5.8)	1.9 (1.7, 2.1)	2.3 (2.0, 2.5)	-0.13 (-0.14, -0.12)
Urbanity						
Rural	1.1 (1.1, 1.2)	1.1 (1.1, 1.2)	1.2 (1.1, 1.2)	0.9 (0.9, 0.9)	0.8 (0.7, 0.8)	-0.01 (-0.02, 0.00)
Urban	1.2 (1.1, 1.2)	1.1 (1.0, 1.2)	1.2 (1.1, 1.3)	0.9 (0.9, 0.9)	0.8 (0.8, 0.9)	-0.02 (-0.02, -0.01)
Metropolitan	1.0 (reference)	1.0 (reference)	1.0 (reference)	1.0 (reference)	1.0 (reference)	1.00 (reference)
Chronic disease (reference=0)						
Stroke	3.4 (3.0, 3.8)	4.5 (4.0, 5.1)	3.5 (3.1, 3.9)	2.1 (1.9, 2.3)	2.0 (1.8, 2.2)	-0.27 (-0.28, -0.25)
Diabetes	1.3 (1.2, 1.4)	1.3 (1.2, 1.5)	1.3 (1.2, 1.4)	1.1 (1.1, 1.2)	1.1 (1.0, 1.2)	-0.05 (-0.06, -0.04)
Hypertension	1.2 (1.1, 1.3)	1.2 (1.1, 1.4)	1.2 (1.1, 1.3)	1.1 (1.0, 1.2)	1.0 (0.9, 1.1)	-0.04 (-0.05, -0.03)
Hyperlipidemia	1.1 (1.0, 1.2)	0.9 (0.8, 1.0)	1.1 (1.0, 1.2)	1.3 (1.2, 1,4)	1.3 (1.2, 1,4)	-0.03 (-0.04, -0.02)
Cardinal	1.5 (1.4, 1.7)	1.4 (1.2, 1.5)	1.6 (1.5, 1.8)	1.5 (1.4, 1.7)	1.5 (1.4, 1.7)	-0.08 (-0.10, -0.07)
No. of chronic diseases						
1	1.0 (reference)	1.0 (reference)	1.0 (reference)	1.0 (reference)	1.0 (reference)	1.00 (reference)
≥2	1.1 (1.0, 1.2)	1.0 (0.9, 1.2)	1.0 (0.9, 1.1)	1.0 (0.9, 1.1)	1.0 (0.9, 1.1)	0.01 (-0.01, 0.02)

All values were calculated by applying sampling weights assigned to individual participants in the survey. aOR, adjusted odds ratio; CI, confidence interval.

1 Odds ratios were adjusted for gender, age group, urbanity, individual income group, occupational, education levels, and number of chronic diseases.

2 The regression coefficient (β) was applied to the marginal effect after controlling for age group, urbanity, individual income group, occupational, education levels, and number of chronic diseases.
